# Editorial: Human factors in transport and road safety

**DOI:** 10.3389/fpsyg.2023.1175488

**Published:** 2023-03-22

**Authors:** Mireia Faus, Francisco Alonso, Angel Egido, Mahdi Rezapour

**Affiliations:** ^1^INTRAS (Research Institute on Traffic and Road Safety), University of Valencia, Valencia, Spain; ^2^Equipe De Recherche 2S2T (Sujets, Sociétés, Territoires, Temporalités), Université Catholique de l'Ouest, Angers, France; ^3^Independent Researcher, Marlborough, MA, United States

**Keywords:** human factor, road safety, mobility, transport systems, development

## Introduction

The development of different transportation systems, especially land transportation, has contributed to the mobility, accessibility and development of societies. Great economic and technological benefits are derived from this flow of goods and people. However, at the same time and together with all the benefits derived from it, the most pernicious aspects appear, such as environmental pollution, visual and acoustic intrusion, congestion and the worsening of habitability in cities, among other elements.

Of all these problems, traffic accidents have the greatest social impact. This problem represents a real pandemic since official figures indicate that every year, about 1,300,000 people die on the roads, and 50 million are injured [World Health Organization (WHO), 2018]. Since numerous researchers agree that the human factor explains approximately 70–90% of accidents, traffic psychology is a fundamental discipline to understanding and working on the behavior of road users.

Consequently, this Research Topic deals with the human factor in the field of traffic, mobility, transportation and road safety, and the relationship with other major factors such as roads, environment, signaling, vehicles and enforcement.

## Main findings of the articles

Flor et al. analyzed the impact of the expansion of new transport services, such as Uber or Cabify, on urban mobility. Specifically, the study aimed to assess whether ridesharing platforms substitute or complement public transport to reduce accident rates, considering the two basic transport zones of Madrid. Data were collected from the 21 districts of Madrid for the period 2013–2019. The results suggest that since the arrival of Uber and Cabify, the number of deaths and serious injuries in traffic accidents has been reduced, as well as the number of traffic accidents on weekends and holidays. However, the number of minor injuries has increased in the central districts of Madrid. In addition, the results suggest that these services are replacing urban buses as an alternative mode of transport, especially for some users.

Yuen et al. investigate how technology and health belief factors influence consumer acceptance of autonomous delivery robots. Five hundred valid responses were collected through an online questionnaire in Singapore, and structural equation modeling was performed to examine the data. The results revealed that perceived ease of use, perceived usefulness, perceived susceptibility, perceived severity, self-efficacy, and cues to action have a positive and significant influence on consumers' perceptions of the value of autonomous delivery robots.

Useche et al. conducted a systematic review to determine the number and type of studies investigating the behavioral perceptions of different groups of road users, contrasting self-reported behavioral data with those reported by other users and their results. The review used the PRISMA methodology, obtaining a final selection of 19 articles that directly address the topic. Thus, it is indicated that road users perceive themselves as “safer” than other road users in terms of their knowledge of traffic rules and their performance on the road.

Grasso and Tagliabue analyzed the relationship between self-reported aberrant behaviors and actual driving performance during a virtual simulation, focusing particularly on speeding. The experiment was conducted on an Italian sample of 79 people who had to drive through virtual road environments. The integrated use of the driving simulator and the Driver Behavior Questionnaire instrument with reference to driving behavior allowed supporting previous theoretical considerations on the relationships between aberrant on-road behaviors and speeding behaviors.

Zhang et al. identify the influence of external factors in promoting customer behavioral intention for electric vehicles. Data from 203 customers in China were analyzed. The results indicated that perceived ease of use and perceived usefulness have a serial mediating relationship between external factors and behavioral intention.

Von Beesten and Bresges studied the effectiveness of a road safety training program applied to high school students (Crash Curs NRW). The results evidenced that a structured follow-up training is adequate to obtain a reactive behavior of the scenic event. The follow-up intervention successfully addressed knowledge deficits about the cause and outcome of crashes.

Antoñanzas and Salavera conducted the validation of the Metacognitive Skills Questionnaire for Drivers of Vehicles (CHMC), which assesses the metacognitive skills used by drivers of vehicles at three points in time (before, during and after driving). The results show the existence of three factors, a planning factor, a self-realization factor and a third evaluation factor.

Cuentas et al. analyzed data from the Naturalistic Engagement in Second Tasks (NEST). The results confirm that the road traffic environment influences distracted driving behavior among automobile drivers; driving uphill compared to driving downhill, in low-density traffic scenarios compared to high-density traffic scenarios, and during afternoon periods compared to morning periods.

## Author contributions

All authors listed have made a substantial, direct, and intellectual contribution to the work and approved it for publication.
